# Interstitial pneumonitis following intrapleural chemotherapy

**DOI:** 10.1186/1477-7819-7-17

**Published:** 2009-02-12

**Authors:** Luis Zappa, Renaldo Savady, Gary N Humphries, Paul H Sugarbaker

**Affiliations:** 1 Program in Peritoneal Surface Malignancy, Washington Cancer Institute, Washington, DC, USA; 2 Grand Valley Medical Specialists, Grand Rapids, MI, USA

## Abstract

**Background:**

Mucinous neoplasms within the abdomen may disseminate by direct extension through the diaphragm to involve the pleural space. Treatment of this condition is by parietal and visceral pleurectomy followed by hyperthermic intrapleural chemotherapy.

**Case presentation:**

In this case report a patient developed persistent right upper lobe interstitial pneumonitis and progressive parenchymal fibrosis following intrapleural chemotherapy treatment with mitomycin C and doxrubicin. The condition persisted until death 28 months later. Death was from progressive intraabdominal disease with intestinal obstruction and sepsis associated with progressive pulmonary parenchymal disease. The right pleural space disease did not recur.

**Conclusion:**

This manuscript is the first case report describing interstitial pneumonitis and lung fibrosis following intrapleural chemotherapy. Since pulmonary toxicity from chemotherapy is a dose-dependent phenomenon, dose reduction of intrapleural as compared to intraperitoneal hyperthermic chemotherapy may be necessary.

## Background

Pseudomyxoma peritonei is the clinical syndrome of mucinous ascites associated with diffuse peritoneal implants of mucin-producing neoplasms [1,2]. It shows varying amounts of invasiveness and histologically the neoplasms producing pseudomyxoma peritonei can be classified as low grade and high grade mucinous carcinomas [3,4]. Appendiceal epithelial cancers represent 1% of colorectal cancers in the USA [5]. Almost all patients with appendiceal mucinous neoplasms have no lymphatic or haematogenous dissemination. Intrathoracic dissemination that occurs from penetration of the diaphragm by the intraabdominal mucinous tumor is rare but does occur in patients with pseudomyxoma peritonei [6]. In the absence of progressive disease in the abdomen, pleurectomy and intrapleural chemotherapy have been used to treat pleural dissemination with excellent clinical results and low morbidity [7]. In this report we present a patient with appendiceal mucinous neoplasm with right pleural dissemination treated by pleurectomy and intrapleural hyperthermic chemotherapy. Acute interstitial pneumonitis was followed by a debilitating long-term fibrosis.

## Case presentation

A 48-year-old woman in early 2004 developed increasing abdominal girth associated with discomfort and pain. A CT scan was performed and a right pelvic mass was visualized. In April 2004, she underwent a debulking procedure which included a right hemicolectomy and omentectomy. Her pathology showed a high grade mucinous epithelial malignancy of the appendix with peritoneal metastases. Because of the extent of disease the patient was referred to the Washington Cancer Institute. In November 2004 she had a 12-hour cytoreductive procedure with hyperthermic (41.5°C) intraoperative intraperitoneal mitomycin C. Early postoperative intraperitoneal chemotherapy with 5-fluorouracil was also used. An end ileostomy was created at the time of her procedure to protect a low colorectal anastomosis and reversed in 2005.

At the time of this operation at our institution, the mid-portions of both right and left hemidiaphragms were resected because of cancer invasion. Visual inspection through the open hemidiaphragm of the pleural space on the left side was clear and remained clear by CT scan. On the right side there were approximately 50 mucinous tumor nodules visualized within the pleural space which were positive by histology for mucinous adenocarcinoma. The patient remained asymptomatic.

**Figure 1:**
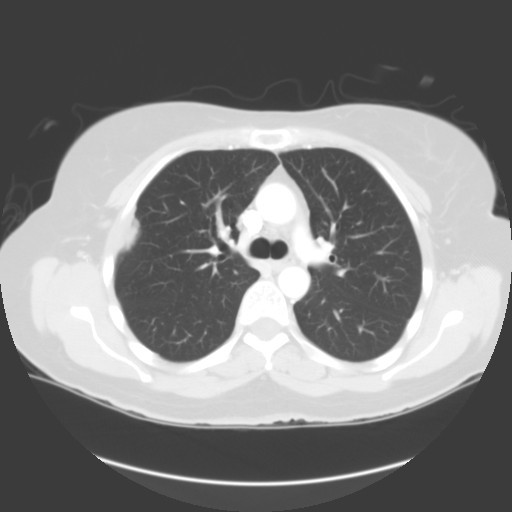
**Chest CT obtained February 2006 showing pleural accumulation of mucinous adenocarcinoma**.

In March 2006 chest CT showed obvious progression of disease in the right pleural space (Figure 1). She had a right thoracotomy with complete stripping of the parietal pleura, partial removal of the visceral pleura and hyperthermic (41.5°) intrapleural chemotherapy with doxorubicin and mitomycin C plus intravenous 5-fluorouracil and leucovorin for 90 minutes. Postoperatively, the patient had an air leak requiring prolonged chest tube drainage. Pathology reported the findings as adenomucinosis [3].

In April 2006 she began to develop progressive dyspnea at rest and increased with exertion. She was unable to take a deep breath without coughing. Her first postoperative CT scan in August 2006 showed minimal visceral cortical scarring on the right with a vague peripheral right mid-lung field opacity.

A CT-guided core needle biopsy of the right upper lobe was performed in April 2007 which demonstrated benign densely fibrotic pulmonary parenchyma with chronic inflammation and entrapped alveolar spaces without evidence of malignancy. The patient was placed on daily prednisone with improvement of her respiratory symptoms. The symptoms remained steroid-dependent with exacerbation on attempted steroid reduction.

**Figure 2:**
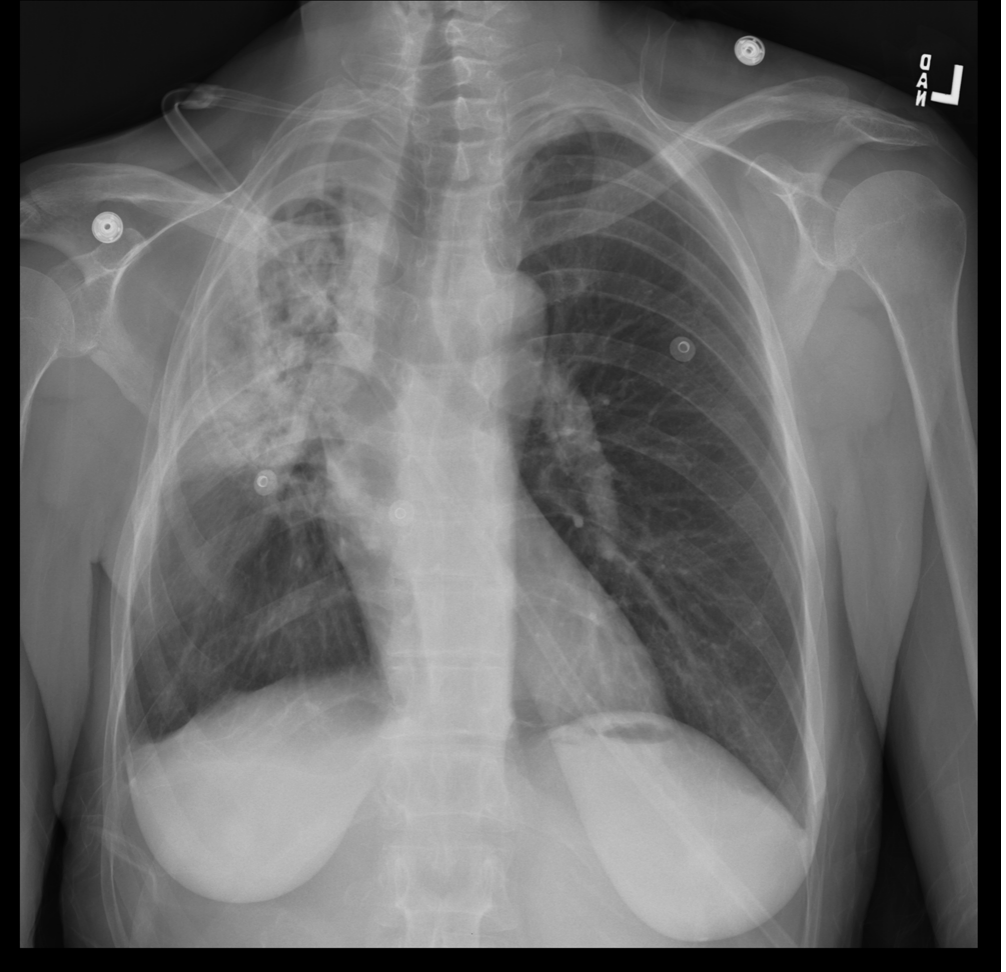
**Chest radiograph obtained April 2008, approximately 2 years after pleurectomy with hyperthermic intraoperative intrapleural chemotherapy**. A persistent contraction from severe fibrosis of the right upper lobe is seen.

**Figure 3:**
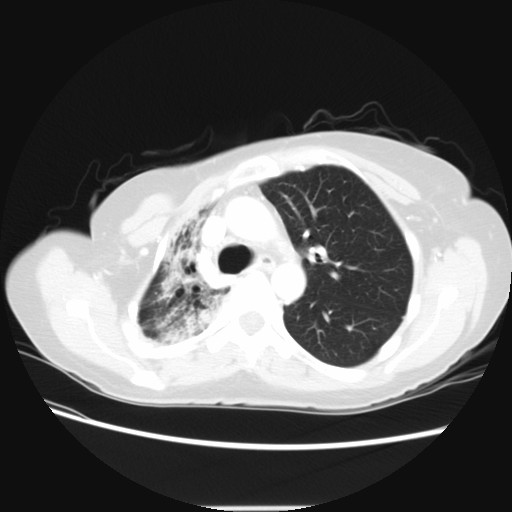
**Chest CT obtained July 2007, approximately 1 year after pleurectomy with hyperthermic intraoperative intrapleural chemotherapy**. An interstitial fibrosis is evident. Subsequent CT are largely unchanged but the patient's symptoms persist.

In early 2008 the patient was functionally performing the normal activities of daily living but had limited respiratory reserve. A persistent cough was present without associated sputum production or fever. A chest radiograph showed contraction and fibrosis of the right upper lobe (Figure 2). A CT scan demonstrated a persistent interstitial fibrosis without evidence of progression (Figure 3). Abdominal CT showed a 1.9 cm × 2.6 cm mass anterior to the superior mesenteric vein associated with an increased CA 19-9 level compatible with progressive intraabdominal disease causing gastric outlet obstruction. No recurrence of disease in the right pleural space was seen. The patient died 28 months after the pleurectomy with progressive fibrosis and infection of the right lung causing sepsis unresponsive to intensive systemic antibiotics. Intestinal obstruction had caused severe nutritional deprivation.

## Discussion

It is known that chemotherapy affects lung function, but it is still unclear to what extent hyperthermic intraoperative intrapleural chemotherapy may cause postoperative complications. Prior to this patient, intrapleural chemotherapy had not been associated with pneumonitis or interstitial fibrosis [7]. An adverse effect on lung function has been documented for many cytotoxic drugs, but the chemotherapy-induced pathologic mechanisms of pulmonary impairment remain unknown [8].

Verweij and colleagues prospectively studied pulmonary interstitial pneumonitis induced by mitomycin C administered by an intravenous route [9]. These authors concluded that mitomycin C-related lung toxicity is a dose-dependent adverse side effect, occurring at cumulative dose levels of 20 mg/m^2 ^or more. The incidence was estimated at less than 10%. Gonzalez-Moreno and coworkers reported a single patient with severe pulmonary interstitial pneumonitis that occurred 37 days after intraperitoneal hyperthermic mitomycin C given at a dose of 50 mg [10]. Oxygen and prolonged oral prednisone led to a full resolution of the symptoms and CT abnormalities after two months of treatment. Gonzalez-Moreno commented that the interstitial pneumonitis they documented was the only case encountered in more than 150 similarly treated patients. They suggested that an idiosyncratic susceptibility may occur with this unusual condition.

When systemic chemotherapy results in lung disease, radiologically the pulmonary damage is always diffuse [11,12]. A normal radiologic study does not exclude the presence of cytotoxic injury, however, it is usually seen. The presence of pleural effusion is unusual with chemotherapy-induced lung disease as a whole but it does not exclude drug toxicity. Purely lobar or segmental density should prompt an alternate explanation. The pulmonary toxicity in our patient was different from prior descriptions in the oncology literature in that intrapleural chemotherapy had caused the damage rather than systemic chemotherapy. Also, the pulmonary damage was localized to the right upper lobe rather than being diffused. We hypothesize that more extensive surgical trauma from visceral pleurectomy at this anatomic site allowed greater localized parenchymal exposure to the sclerotic effects of mitomycin C and doxorubicin.

The second local-regional chemotherapy agent used in the pleural space of this patient was low-dose doxorubicin. This drug diluted in 3 liters of chemotherapy solution has been shown to be well tolerated in the peritoneal cavity and has been used in more than 400 patients [13]. However, for both intrapleural mitomycin C and for intrapleural doxorubicin, an increased risk of local-regional toxicity may exist. As the hyperthermia lavage of the lung progresses over 90 minutes, the intrapleural drugs are slowly cleared into the systemic circulation. If even minute amounts of chemotherapy enter the alveolar spaces of the lung, this may greatly amplify the toxicities caused by drugs present in the blood. The case report of Gonzalez-Moreno confirmed that the systemic effects of intraperitoneal chemotherapy can cause pulmonary interstitial pneumonitis [10].

Diffuse pulmonary disease has been seen in patients receiving chemotherapy. There may be a wide variety of etiologies which include pulmonary infection, complications of the underlying disease and direct toxicity from cytotoxic drugs. The clinical syndromes of pneumonitis and fibrosis have a biphasic pathological course and are dependent upon the dose and volume of lung exposed. The pulmonary complications following systemic chemotherapy can be acute in onset or may develop insidiously months or years after treatment. Usually the clinical syndrome occurs 1–3 months after completion of the drug therapy. The severity of symptoms of the acute pneumonitis syndrome is dependent on the degree of pulmonary involvement. It progresses eventually to a fibrotic phase [12]. The mainstay of treatment has been steroids.

Although unusual, thoracic involvement by pseudomyxoma peritonei of appendiceal origin has been well documented as pleural effusions or pulmonary metastasis [7,14]. The time frame between initial diagnosis of pseudomyxoma peritonei and the discovery of thoracic disease related to this syndrome ranged from less than 1 year to nearly 15 years. The average period of time, however, is between 2 and 6 years [7]. There are several mechanisms which have been proposed in order to explain how neoplastic cells can spread to the pleural space from the peritoneal cavity: (1) iatrogenic perforation of the diaphragm while a subdiaphragmatic peritonectomy is performed; (2) incidental finding, when the pleural space is entered as in the case presented; (3) dissemination via direct invasion or through lymphovascular spaces of the diaphragm; (4) and in a small proportion of cases, congenital or acquired pleuroperitoneal communications which allow neoplastic cells to reach the pleural spaces [15].

The cytologic and histologic features of the metastasis resemble the primary appendiceal neoplasm [1,3]. As the diagnosis of pseudomyxoma peritonei is usually made early in the patient's history, the differential diagnosis from the thorax should not pose a great challenge. The presence of intracytoplasmic and extracellular mucin should lead to the correct diagnosis. Mucinous material is often abundant in the background of very few cancer cells. An aspiration cytology of tumor in the pleural space may show acellular mucin or the tumor cells may be surrounded by large volumes of mucin and difficult to positively identify.

## Conclusion

From our review of this patient's clinical course and our experience with other patients who had the same procedure, the causation of this interstitial fibrosis is not apparent. Although parenchymal damage to the lung is well described from chemotherapy, a localized and persistent fibrosis of lung parenchyma as seen in this patient given intrapleural chemotherapy has not been previously reported. This localized pneumonitis which progressed to fibrosis and then systemic sepsis was likely related to combined systemic and local-regional toxic effects of chemotherapy. It is likely that visceral pleurectomy prior to the hyperthermic intrapleural chemotherapy treatment could result in access of small amounts of drug solution to lung parenchyma. The multi-agent chemotherapy used for this pleural extension of adenocarcinoma contains vesicant drugs that could cause fibrosis to the delicate lung parenchyma. Theoretically, surface treatment should have no parenchymal toxicity; however, damage to lung parenchyma by visceral pleurectomy may allow tissue penetration by chemotherapy. Progressive lung inflammation and fibrosis may result in a long-term disability and contribute to the demise of the patient.

## Abbreviations

CT: computed tomography; CA 19-9: cancer antigen 19-9

## Competing interests

The authors declare that they have no competing interests.

## Consent

Written informed consent was obtained from the patient's next of kin for publication of this case report and accompanying images. A copy of the written consent is available for review by the Editor-in-Chief of this journal.

## Authors' contributions

All authors made substantial contributions to the concept, design, acquisition of data, analysis and interpretation of data, drafting and revising the intellectual content of the manuscript. All authors read and approved the final manuscript.
